# Multiple roles of baicalin and baicalein in the regulation of colorectal cancer

**DOI:** 10.3389/fphar.2024.1264418

**Published:** 2024-02-05

**Authors:** Jiamei Wang, Zihong Wu, Jiayuan Peng, Fengming You, Yifeng Ren, Xueke Li, Chong Xiao

**Affiliations:** ^1^ Hospital of Chengdu University of Traditional Chinese Medicine, Chengdu, China; ^2^ Institute of Oncology, Chengdu University of Traditional Chinese Medicine, Chengdu, China; ^3^ Oncology Teaching and Research Department of Chengdu University of Traditional Chinese Medicine, Chengdu, China

**Keywords:** baicalin, baicalein, colorectal cancer, apoptosis, signaling pathways

## Abstract

The prevalence of colorectal cancer is increasing worldwide, and despite advances in treatment, colorectal cancer (CRC) remains in the top three for mortality due to several issues, including drug resistance and low efficiency. There is increasing evidence that baicalin and baicalein, novel small molecule inhibitor extracts of the Chinese herb Scutellaria baicalensis, have better anti-colorectal cancer effects and are less likely to induce drug resistance in cancer cells. The present review article explains the anti-proliferative properties of baicalin and baicalein in the context of against CRC. Additionally, it explores the underlying mechanisms by which these compounds modulate diverse signaling pathways associated with apoptosis, cell proliferation, tumor angiogenesis, invasion, metastasis, and tumor microenvironment. Moreover, this review article highlights the inhibitory effect of colorectal inflammatory-cancer transformation and the near-term therapeutic strategy of using them as adjuvant agents in chemotherapy.

## 1 Introduction

The incidence rate of colorectal cancer (CRC) has been steadily increasing at a rate of 10% per year with the changes in people’s living environment and dietary habits, making it the third most prevalent malignant tumor following lung cancer and breast cancer (The, 2017). According to the latest data, colon cancer cases more than doubled from 1990 to 2019, and rapid growth is particularly evident in countries with low-to-moderate socio-demographic indices in Asia and Africa (GBD, 2019 Colorectal Cancer Collaborators, 2022). It is now the second leading cause of cancer-related deaths, with a mortality rate of 9.4%. Additionally, there has been a marked trend towards a younger age group for this disease in recent years and Significant increase in the incidence of colorectal cancer before the age of 50 years, especially in countries with high socio-demographic populations (Dekker et al., 2019; GBD, 2019 Colorectal Cancer Collaborators, 2022; Sinicrope, 2022). The 5-year survival rate of CRC patients is only 20% (Biller and Schrag, 2021). Furthermore, the 5-year survival rate after the occurrence of distant metastases is less than 10%. These figures significantly contribute to a 60% increase in the economic burden on society and pose a substantial threat to the global population’s life and wellbeing (Ge et al., 2022). Currently, several therapeutic interventions for CRC exist, including surgical resection, systemic chemotherapy, local radiotherapy, and immunotherapy. The existence of various treatments for CRC is accompanied by certain drawbacks, including drug resistance, bone marrow suppression, and gastrointestinal reactions, Moreover, these treatments fail to adequately address the issues of high clinical morbidity and mortality associated with CRC, Consequently, there is a growing demand within the clinical community to identify novel small molecule inhibitors that can effectively intervene in CRC.

The value of Chinese medicine in treating CRC has recently received increasing attention. Several studies have shown that certain components of herbal medicine have chemopreventive and adjuvant chemotherapeutic potential with low toxic side effects and high safety and have achieved remarkable results in improving the quality of survival and survival rate of patients (Xiang et al., 2019). Baicalin (C_21_H_18_O_11_) and baicalein (C_15_H_10_O_5_) are the primary bioactive constituents found in the dried rhizome of Scutellaria baicalensis, a traditional Chinese medicinal plant (Hu et al., 2022). These compounds are classified as flavonoids and can be isolated using various techniques such as precipitation and washing, high-performance liquid chromatography (HPLC), ultrafiltration membrane separation, and other similar methods, The two compounds are structurally similar and can be interconverted in the body after metabolism in the human intestine. Moreover, baicalein can be combined with one molecule of glucuronide by glucuronosyltransferase (UGT) to produce baicalin, while baicalin is reduced to baicalein by glucuronosidase (GUS) hydrolysis (Yun et al., 2022). Previous research studies have confirmed that both compounds have various clinical applications due to their properties of anti-inflammatory, antibacterial, antiviral, anti-cardiovascular, and anti-neurodegenerative diseases (Ran et al., 2021; Chen et al., 2022; Wen et al., 2023). Recent evidence shows that baicalin and baicalein play an important role in inducing cell death, inhibiting cell proliferation, blocking tumor blood vessel formation, inhibiting cell invasion, and metastasis, as well as regulating the tumor microenvironment (Wang et al., 2022a). The compounds possess diverse mechanisms of action against tumors, exhibiting multi-pathway, multi-level, and multi-target effects. For instance, they have been observed to induce apoptosis in lung and breast cancer and modulate adhesion signaling in ovarian cancer cells (Ma et al., 2020; Park et al., 2021; Zheng T. et al., 2023). Consequently, these compounds hold promise as novel therapeutic agents for CRC treatment.

The mechanism of action of baicalin and baicalein against CRC has not been comprehensively examined. Therefore, this article reviews and discusses the potential molecular mechanisms and related signaling pathways of these two novel small molecule inhibitors against CRC. Additionally, it explores the feasible strategies for baicalin and baicalein to inhibit colorectal inflammation-cancer transformation and adjuvant chemotherapy. Furthermore, it sheds light on the potential avenues for future investigation in this field. The objective of this study is to facilitate the clinical application of baicalin and baicalein in the context of colorectal cancer, with the aim of identifying potential therapeutic interventions for the treatment of this disease. Additionally, this research aims to explore strategies for enhancing the overall quality of survival and long-term outcomes for patients affected by colorectal cancer.

## 2 Baicalin and baicalein mediated anti-colorectal cancer efficacy

### 2.1 Inducing CRC cell death

Several studies have suggested that programmed cell death (PCD) plays an important role in the fight against CRC and can occur through specific molecular pathways. Conversely, these pathways can be controlled by pharmacological or genetic means (Peng et al., 2022; Yu et al., 2022). In contrast, baicalin and baicalein can induce CRC cell death through apoptosis, cell autophagy, and cell necrosis (Green and Llambi, 2015).

#### 2.1.1 Induction of apoptosis

In the regulation of apoptosis in humans, when the expression of pro-apoptotic proteins, including the Bcl-2 family of proteins and IAPs, is greater than that of anti-apoptotic proteins, the endogenous mitochondrial apoptotic pathway is initiated. This leads to the release of mitochondrial outer membrane permeabilization (MOMP) and thus cytochrome C (Cyt C), eventually forming a complex with the apoptosis protease activator Apaf-1 to recruit caspase-9 and protein hydrolysis. Ultimately, this cascade activates downstream caspase-3 culminating in the execution of apoptosis (Chandrashekar et al., 2012; Zheng et al., 2016). The apoptotic cell count of CRC cells increased significantly after baicalin and baicalein treatment. The researchers conducted microscopic observations and found that the presence of baicalin and baicalein in CRC cells led to the formation of densely packed chromatin and the fragmentation of nuclei. Simultaneously, a significant loss of mitochondrial membrane potential and upregulation of ROS levels were detected (Lai et al., 2015). It was found that the expression of XIAP, Bcl-2, Bcl-29, and NF-kB, which inhibited apoptosis, decreased, while the expression of caspase-3, caspase-9, and Parp-1, which promoted apoptosis, increased (Lai et al., 2015; Bai et al., 2017; Tao et al., 2018; Song et al., 2022). These findings confirm that baicalin and baicalein possess anti-apoptotic properties in CRC by modulating the equilibrium between apoptosis-inhibiting and apoptosis-promoting proteins.

The administration of baicalin and baicalein to CRC cells led to the aggregation of nuclei, their confinement, and the formation of apoptotic vesicles. Simultaneously, a concentration and dose-dependent increase in TP53, TP53BP2, tumor necrosis factor (TNF), and downstream caspase-3, caspase-8, and caspase-9 activities were detected. Moreover, a downregulation of TNFRSF10B expression, which in turn initiated the exogenous death receptor pathway to cause apoptogenesis (Wang et al., 2013; Wang et al., 2019). In addition to the above results detected in some studies, the treatment of CRC cells resulted in nuclei agglutination, sequestration, and formation of apoptotic vesicles of the nuclei, as well as a concentration and dose-dependent increase in the activity of necrosis factors TNFTP53, TP53BP2, TNF and downstream caspase-3, caspase-8, and caspase-9, which were detected. The expression of TNFRSF10B was downregulated. This suggests that in addition to the endogenous mitochondrial apoptotic pathway, the exogenous death receptor pathway plays an important role in anti-colorectal cancer effects of baicalin and baicalein. In a separate investigation, it was observed that the levels of phosphorylation in caspase-3, caspase-8, caspase-9, and Parp-1 exhibited an increase subsequent to the administration of baicalin. In contrast, there was no significant alteration observed in the levels of P53 protein, and it is speculated that baicalin may also induce apoptosis in colorectal cancer cells through a non-P53-dependent pathway (Yang et al., 2020). Due to the development of artificial intelligence computer technology in medicine, researchers have discovered through computer modeling that baicalein forms hydrogen bonds with residues Leu227 and Asp228 of caspase-9 through hydroxyl groups and with residues Ser251 and Asp253 of the caspase-3 active site, thereby enhancing the execution of the apoptotic program initiated by both activities (Wang et al., 2015).

The initiation of these apoptotic mechanisms can be regulated by a variety of signaling pathways as well as by genes. The mRNA expression and phosphorylation levels of PI3K, AKT, and GSK-3β can affect the expression of Gli1 and other molecules downstream of the Hedgehog signaling pathway (Jia et al., 2019; Liu, 2020). The mRNA expression and phosphorylation levels of PI3K, AKT, and GSK-3β were inhibited by baicalin treatment in CRC, causing apoptosis of CRC cells. However, this effect was attenuated using the pathway inhibitor (LY294002) (Li et al., 2021). Simultaneously, a decrease in the expression of SHH, SMO and Gli1-related mRNA was detected, and an increase in the expression of SUFU mRNA, suggesting that it is anti-colorectal cancer effect may be achieved by affecting the PI3K/AKT/GSK-3β and Hedgehog signalling pathways (Lin H. D. et al., 2023). MET upregulation increased TOPK expression and thus activated AKT to promote colon carcinogenesis. Treatment with baicalein detected upregulation of activated caspase-3 and PARP expression and downregulation of MET, AKT, and H3 phosphorylation levels in cells. Conversely, phosphorylated H3, a TOPK metabolite, was presumed to play an apoptotic role by inhibiting the MET/AKT signaling pathway (Xu et al., 2022). The Wnt/β-catenin signaling pathway is associated with apoptosis, invasion, and cell proliferation resistance, and its aberrant activity is associated with the progression of CRC (Wang et al., 2015). The Wnt/β-catenin pathway affects the expression of downstream c-Myc genes and regulates the family of Bcl-2 proteins. The DKK1 protein is an important Wnt pathway antagonist, it can inhibit the Wnt pathway through LRP5/6 and microRNA-217 can reduce DKK1 expression. Baicalin was found to downregulate microRNA-217 and thus upregulate DKK1 in CRC cells, inhibiting the expression levels of mRNA and proteins of the downstream molecules β-catenin and c-Myc, causing apoptosis (Jia et al., 2019; Liu, 2020). In another study, baicalein was found to upregulate DEPP and Gadd45a expression and promote MAPK phosphorylation. This effect was diminished by inhibition of the downstream signaling pathway JNK/p38 but not by inactivation of extracellular signal-regulated kinases, suggesting that baicalein induces a positive feedback loop between Gadd45a and JNK/p38 to promote the activation of MAPKs through the upregulation of DEPP, leading to an apparent apoptotic response in human colon cancer cells. Baicalin and baicalein can act directly on gene expression and affect downstream gene expression through signaling pathways. Moreover, they can selectively activate ERK1/2 and p38 and regulate MAPK/p38 and ERK1/2 signaling pathways to induce apoptosis in CRC cells (Su et al., 2018). Inhibition of the transcription factor Sp1 induces apoptosis in colon cancer stem cells (Zhao et al., 2013). Downregulation of Sp1 expression after baicalin treatment was found to increase downstream caspase-3 and PARP protein expression. Caspase-3 can cause shearing of the PARP active region, resulting in loss of enzymatic activity leading to cell instability and promoting the execution of an apoptotic program (Ma et al., 2019). Additionally, the inflammation-stimulating factor COX-2 increased in many tumors, including colon, breast, and prostate cancer (Stasinopoulos et al., 2013; Fenner, 2016; Su et al., 2016). Several studies have shown that COX-2 inhibitors can inhibit the growth of CRC cells and induce apoptosis. Moreover, experimental evidence has confirmed that baicalin can downregulate the COX-2 protein to play a similar inhibitor effect and promote apoptosis in CRC cells (Wu et al., 2016).

**FIGURE 1:**
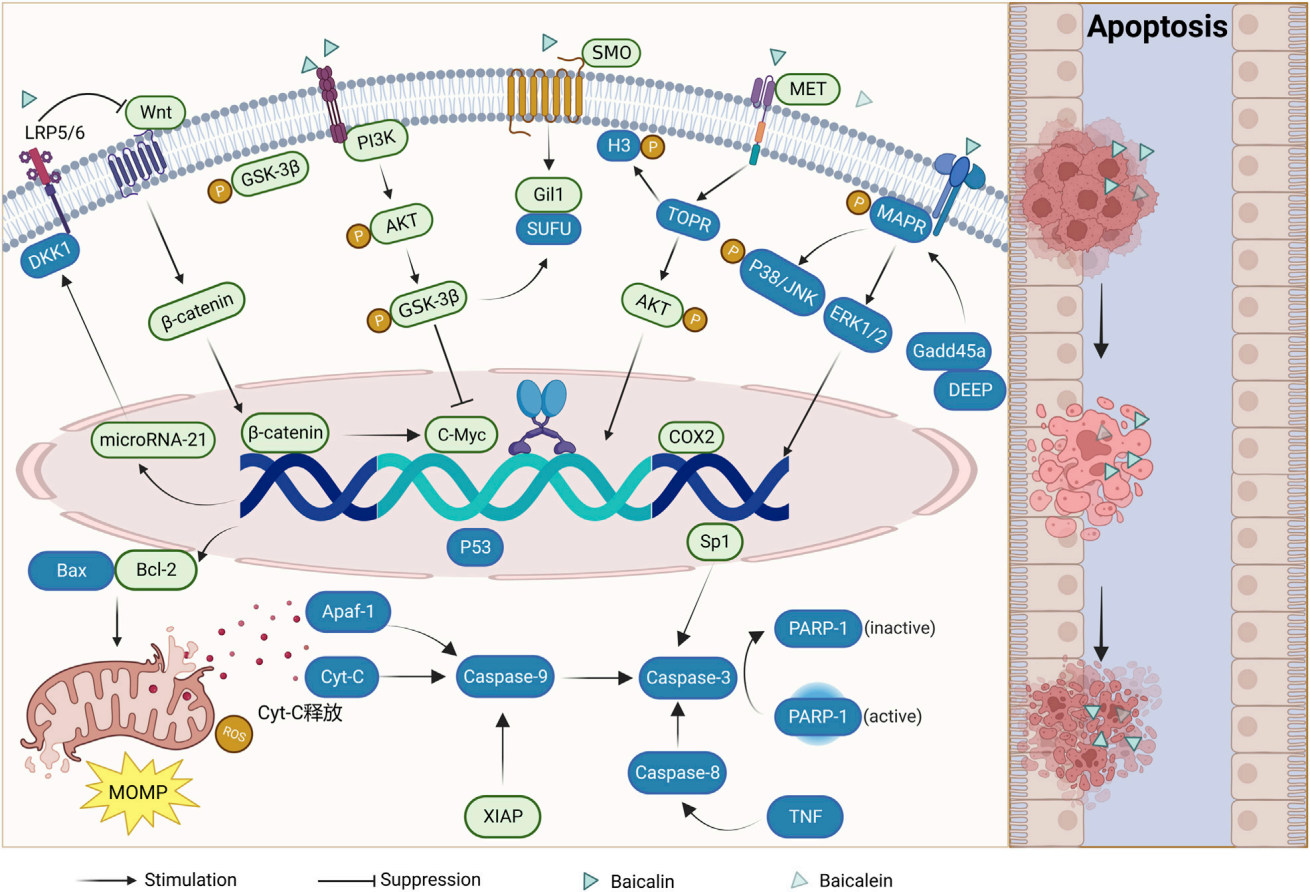
Mechanism of apoptosis induction by baicalin and baicalein (Boxes in green indicate downregulation of expression and boxes in blue indicate upregulation of expression).

Although the signaling pathways and molecular mechanisms that trigger apoptosis are different, baicalin and baicalein promote apoptosis as an anti-colorectal cancer effect. This is achieved by regulating various molecular signaling pathways and genes, causing an imbalance between pro-apoptotic and anti-apoptotic pathways, including the Bcl-2 protein family and IAPs. Consequently, this imbalance triggers the activation of endogenous mitochondrial apoptotic pathways or exogenous apoptosis mediated by TNF. The Caspase family plays an important role in the whole process. (As shown in Figure 1).

#### 2.1.2 Regulating autophagy

Autophagy refers to the cellular process wherein cytoplasmic proteins or organelles are engulfed, enclosed within vesicles, and subsequently fused with lysosomes (Biazik et al., 2015). This fusion results in the formation of autophagic lysosomes, which play a crucial role in the degradation of the enclosed contents. This mechanism is of significant importance in the maintenance of cellular material turnover. Recently, it has been found that promoting cellular autophagy can be a new avenue for tumor treatment (Debnath et al., 2023). Evidence suggests that upregulation of autophagy proteins such as LC3II and Beclin1 can be detected after baicalin and baicalein act on CRC cells, suggesting both may exert anti-colorectal cancer effects by promoting cellular autophagy and inducing apoptosis (Tan et al., 2019; Phan et al., 2020).

#### 2.1.3 Promoting CRC cell necrosis

Unlike apoptosis, necrosis is the death of localized tissue cells in the living body, characterized by changes in enzymatic solubility. Necrosis can result directly from strong causative factors, but most develop from reversible damage, the basic manifestations of which are cell swelling, organelle disintegration, and protein denaturation. A previous study observed by transmission electron microscopy (TEM) that baicalin treatment of CRC cells resulted in densely stained nuclei, nuclear fragmentation, mitochondrial swelling, and contents leakage. Simultaneously, the expression product of the RIP3 gene, a key protein in the induction of cancer cell necrosis, was significantly increased. This indicates that baicalin may play an anti-colorectal cancer role by inducing necrosis while inducing apoptosis (Yang et al., 2019).

The above evidence confirms that baicalin and baicalein do not cause CRC cell death through a single pathway. However, apoptosis induction, autophagy promotion, and necrosis induction all play a role in the anti-colorectal cancer process of both.

### 2.2 Inhibition of CRC cell proliferation

#### 2.2.1 Cell cycle arrest

**FIGURE 2:**
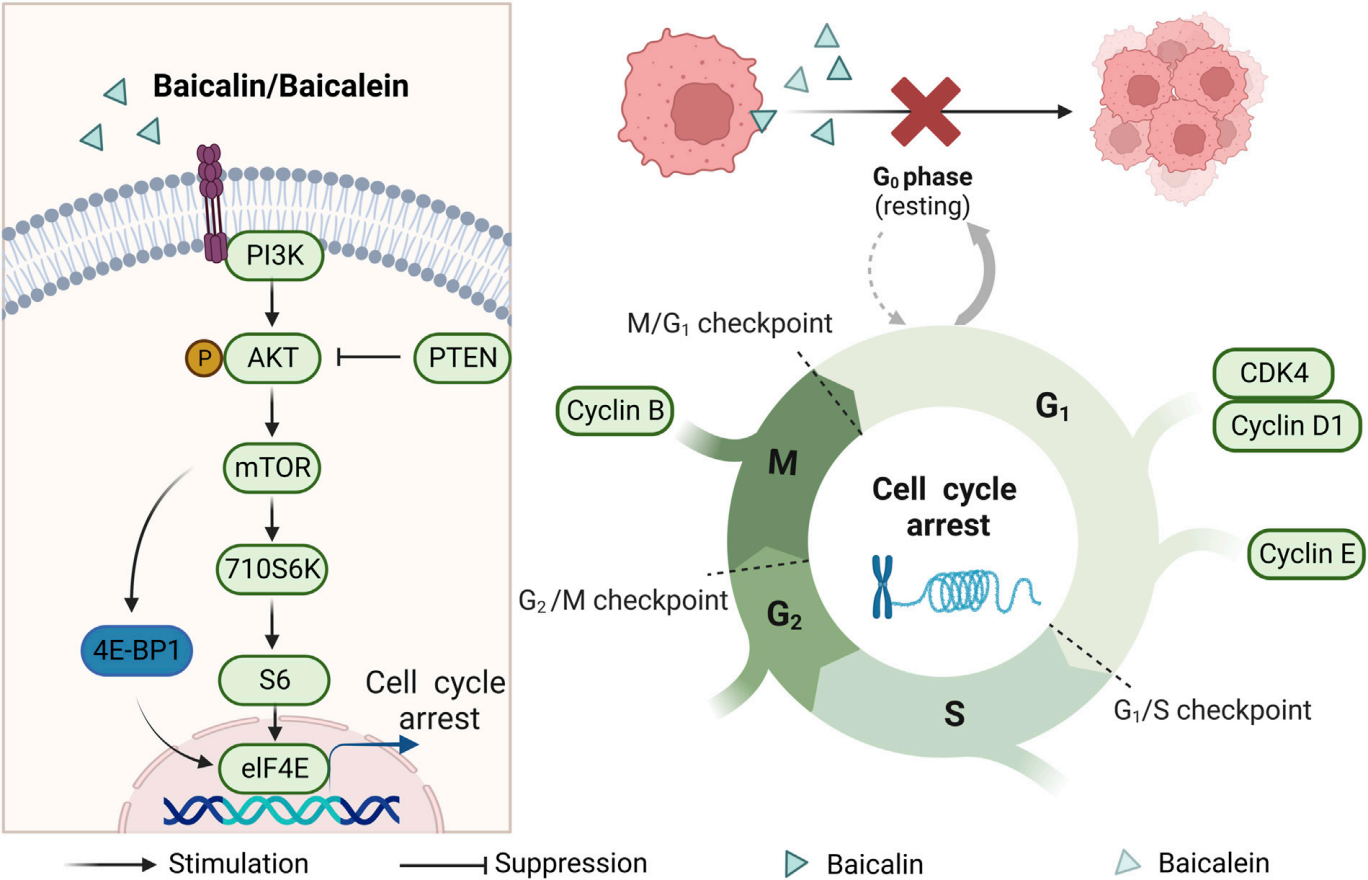
Mechanism of cell cycle blockade by baicalin and baicalein.

The cell cycle is closely related to cell proliferation, and one of the reasons for tumorigenesis is the unregulated cell cycle (Mohammad et al., 2015). Several previous studies have found that the proportion of CRC cells in phases G1 and S increased after baicalin and baicalein treatment. Moreover, the expression of cyclinD1/E1/B1, p-AKT (Ser473), Ezrin, and CDK4, which are related to the cell cycle, was reduced. It was shown that baicalin and baicalein could block the cell cycle of CRC cells in the G1 phase to block cell proliferation (Chen et al., 2012; Kim et al., 2012; Wang et al., 2013; Bai et al., 2017; Chen et al., 2018; Dou et al., 2018; Yang et al., 2020). PCNA is closely related to cellular DNA synthesis, plays an important role in the initiation of cell proliferation, and is a good indicator of the proliferation status of cells. Baicalein was found to reduce the expression of PCNA in CRC cells (Palko-Labuz et al., 2017). As the study progressed, the mTOR signaling pathway was found to be over-activated in tumor cells. Meanwhile, a decrease in AKT expression was observed following treatment with baicalin and baicalein. Additionally, the expression of mTOR, p70S6K, S6, and eIF4E was downregulated, while the expression of 4E-BP1 was upregulated. These findings indicate that both baicalin and baicalein exhibit anti-colonial effects by inhibiting the mTOR pathway. Consequently, the enzymatic activity necessary for the cell cycle is reduced, leading to cell cycle arrest (Wu et al., 2012; Wang et al., 2019) (As shown in Figure 2).

#### 2.2.2 Reducing CRC cell stemness

Cell stemness is important in metastasis, drug resistance, and tumor cell recurrence (Jin et al., 2017; Muinao et al., 2018). Baicalin inhibited the formation of CRC stem cell spheres in a concentration-dependent manner, and reduced levels of stem cell marker proteins such as CD44, CD2, SOX4, OCT4, and Nanog were detected, suggesting that baicalin may inhibit stem cell development and thus CRC cell proliferation (Yang et al., 2020). Further study revealed that the expression levels of SHH, SMO, and Gli1-related proteins were inhibited, and the expression level of SUFU protein was increased in CRC cells after baicalin treatment. These findings suggest that baicalin is crucial for inhibiting cell growth by down-regulating tumor cell stemness through inhibiting the Hedgehog pathway in CRC cells (Lin H. D. et al., 2023).

### 2.3 Blocking tumor angiogenesis

**FIGURE 3:**
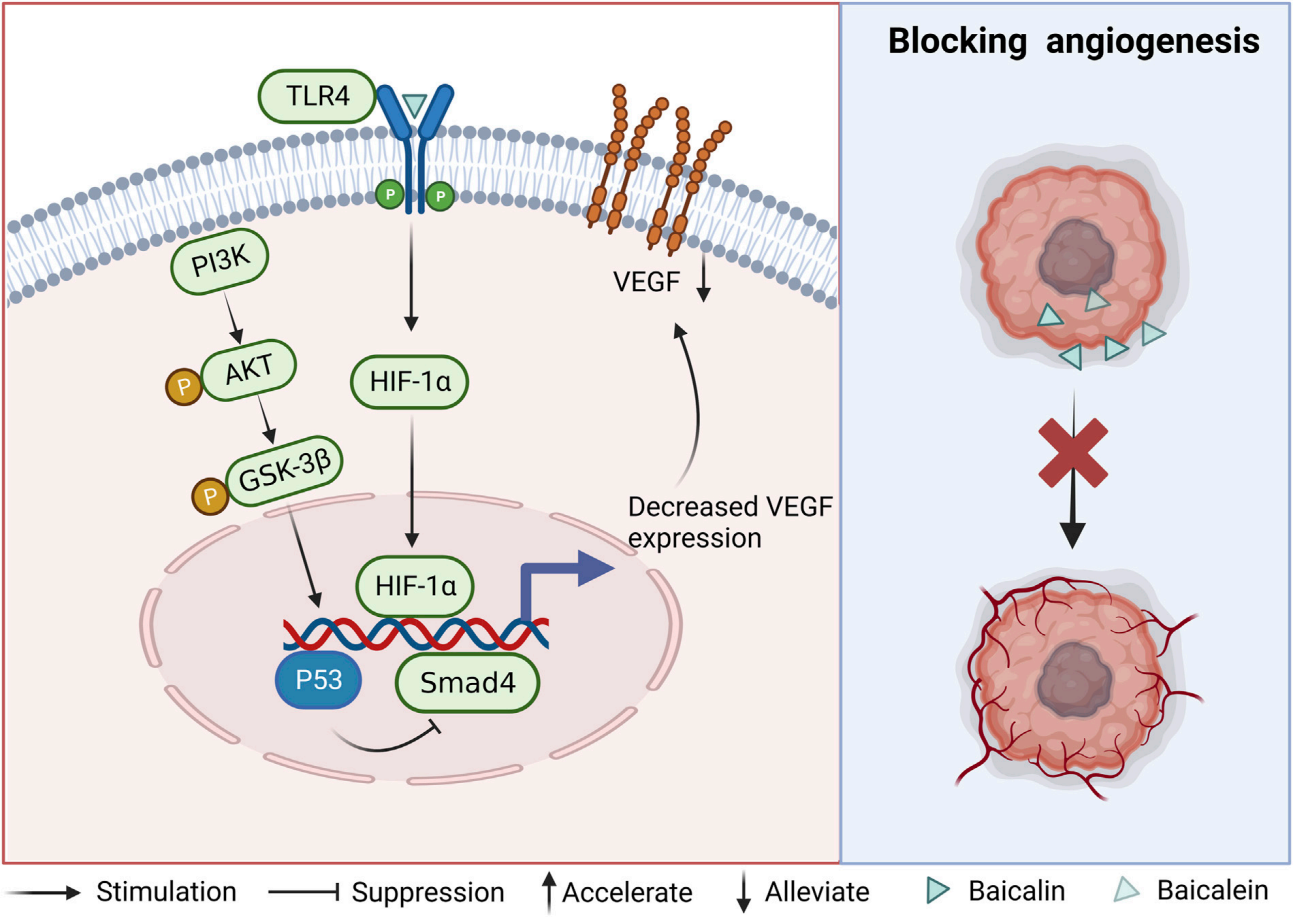
Mechanism of angiogenesis inhibition by baicalin and baicalein.

The role of vascular endothelial growth factor (VEGF) in tumor growth has been demonstrated, highlighting its significance as a pivotal vascular factor in the progression of tumors (Apte et al., 2019). The administration of baicalein was observed to induce upregulation of the oncogenic protein p53, resulting in the suppression of the angiogenic gene Smad4 and subsequent inhibition of VEGF formation in CRC cells (Wang et al., 2019). Another study has shown that the expression of VEGF, HIF-1α, and CD31 was downregulated after the administration of baicalein. However, the effect of baicalein disappeared after knocking out TLR4. In contrast, baicalein was found to bind to TLR4 and form a blocking complex (LPS.MD-2. TLR4) 2 to inhibit TLR4 activity, thereby reducing the expression of downstream molecules HIF-1a and VEGF. This evidence suggests that baicalein may act through the TLR4/HIF-1α/VEGF signaling pathway (Chen et al., 2021). Reduced angiogenesis was observed after baicalin treatment of CRC cells. It was accompanied by the downregulation of PI3K, AKT, and GSK-3β mRNA, However, this decrease did not reach statistical significance upon the introduction of LY294002, a PI3K pathway inhibitor. Presumably, baicalin inhibits the PI3K/AKT/GSK-3β pathway (Li et al., 2021). The above evidence demonstrates that baicalin and baicalein can interfere with VEGF expression through different signaling pathways, inhibit angiogenesis and thus block the growth and progression of CRC cells (As shown in Figure 3).

### 2.4 Inhibition of CRC cell invasion and metastasis

#### 2.4.1 Inhibition of epithelial-mesenchymal transition (EMT)

Tumor invasion and metastasis is a very complex process in which the degradation of the extracellular matrix and the basement membrane is a key step in the epithelial-mesenchymal transition (EMT). This transition subsequently induces alterations in cellular morphology, loss of adhesion, and heightened acquisition of stem cell-like traits, ultimately culminating in the metastatic dissemination of tumor cells. The researchers detected increased expression of epithelial markers such as E-cadherin, Cytokeratin18, and Claudin1 and decreased expression of mesenchymal markers such as snail, N-cadherin, and Vimentin. These results suggest that baicalin and baicalein exert their anti-colorectal cancer effects by inhibiting the degradation of the extracellular matrix and thus blocking the development of the EMT (Yang et al., 2020; Zeng et al., 2020). With the development of the study, researchers have gradually explored the molecular mechanism of regulation of the EMT and found that various signaling pathways, such as TGFβ and Wnt, play key regulatory roles in the EMT (Gonzalez and Medici, 2014). After baicalin treatment of CRC cells, reduced levels of endogenous protein expression and phosphorylation of TGF-1, Smad2/3, and Smad4 along with elevated of endogenous TGFβ1, Smad2/3, and Smad4, and increased protein expression of Smad7 were detected, indicating that baicalin inhibition of the EMT of cells may be associated with suppression of the TGFβ/Smad signaling pathway in CRC cells (Yang et al., 2020). In another study, the expression of miR-217 in CRC cells was downregulated by baicalin treatment. Conversely, the expression levels of DKK1, a key negative regulator of the downstream Wnt signaling pathway, increased, suggesting that baicalin could exert its invasive and anti-tumor migratory effects by inhibiting the activation of small molecules miR-217 that regulate the Wnt signaling pathway (Liu, 2020).

**FIGURE 4:**
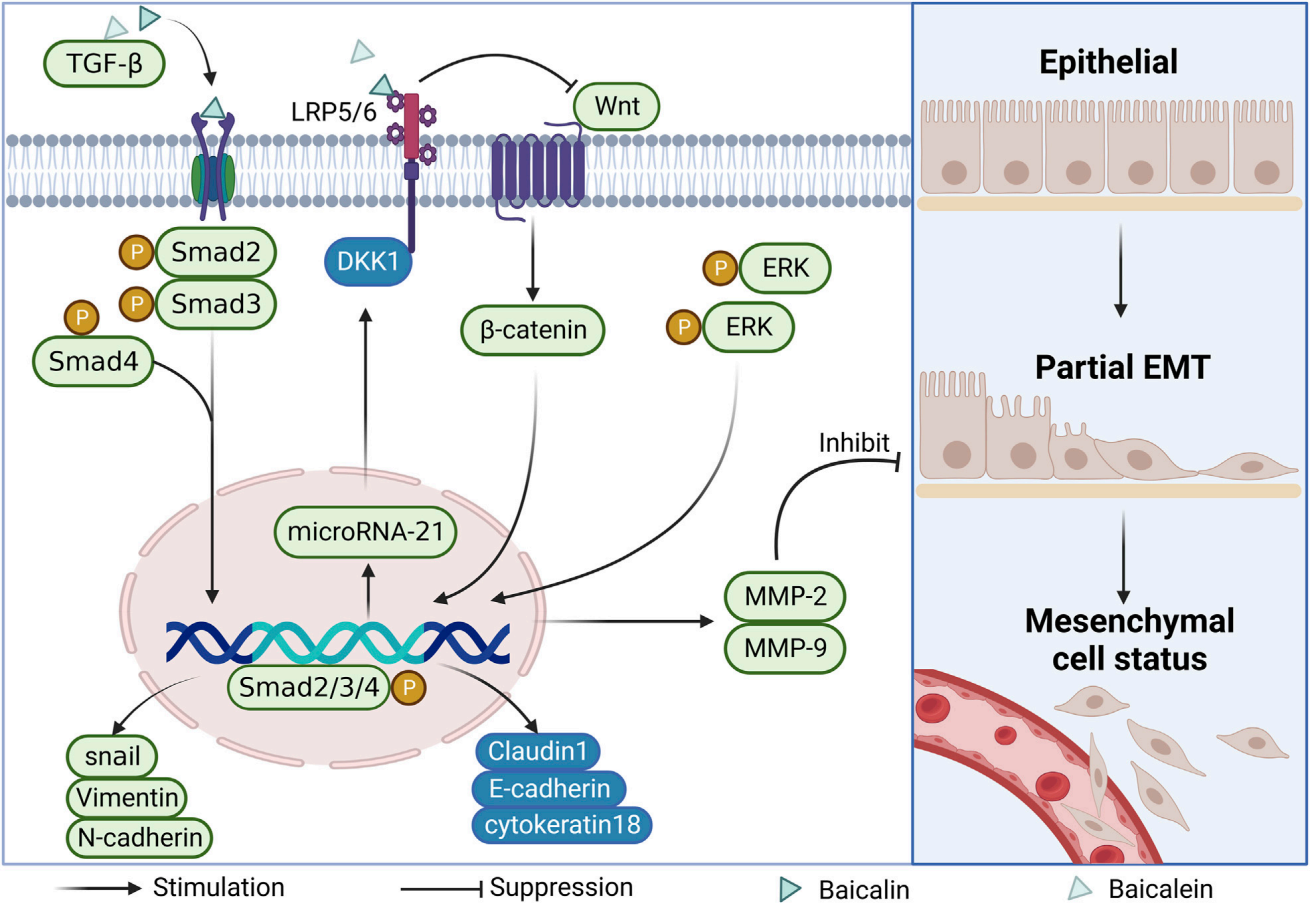
Mechanism of EMT inhibition by baicalin and baicalein.

Matrix metalloproteinases (MMPs) are a family of enzymes with zinc-binding activity that degrade the extracellular matrix. MMPs can degrade the extracellular matrix and basement membrane, and their stability is closely related to tumor metastasis, especially, the high expression of MMP-2 and MMP-9 plays an important role in tumor growth and metastasis (Löffek et al., 2011). Related evidence showed that the expression of invasion and activation signaling molecules such as MMP-2 and MMP-9 were significantly downregulated after baicalin and baicalein acted on CRC cells (Kim et al., 2013; Wu et al., 2016). A further study revealed that the upstream signaling pathway ERK phosphorylation level was reduced and the expression of MMP-2 and MMP-9 was downregulated after baicalein action. These findings suggest that inhibiting the ERK signaling pathway may be a key molecular mechanism for baicalein to reduce the invasion of CRC (Chai et al., 2017) (As shown in Figure 4).

#### 2.4.2 Inhibition of CRC cell aggregation

In addition to EMT, tumor cell aggregation and migration play an important role in tumor metastasis. Further comparison revealed that the number and size of colonies formed by CRC cells were significantly reduced after treatment with baicalin and baicalein (Patel et al., 2018). Additionally, the migration ability was reduced, and the inhibitory effect of baicalin on migration was not as obvious as baicalein. This disparity in efficacy may be attributed to the different molecular structures of these two compounds (Dou et al., 2018). Signaling pathways also regulate cell aggregation and migration. Activation of the NF-κB and AKT pathways is closely related to tumor progression and distant metastasis. In CRC, increased levels of protein and phosphorylation in the AKT pathway are important ways to promote tumor growth and metastasis (Patel et al., 2018; Liu et al., 2019). It was found that the expression levels of TLR4, NF-κB, p65, and p-IκBα were significantly downregulated in CRC cells after baicalin treatment, and the inhibitory effect was diminished after the use of TLR4 activator, indicating that baicalin could lead to impairment of TLR4/NF-κB signaling pathway and thus inhibit the migration and invasion of CRC cells (Song et al., 2022). Another study found that the levels of protein phosphorylation of the PI3K, AKT, and GSK-3β protein phosphorylation levels decreased after baicalin treatment, suggesting that inhibition of the PI3K/AKT/GSK-3β pathway also plays an important role in the inhibition of CRC cells migration by baicalin (Liu et al., 2019; Li et al., 2021).

### 2.5 Regulation of the tumor microenvironment (TME)

**FIGURE 5:**
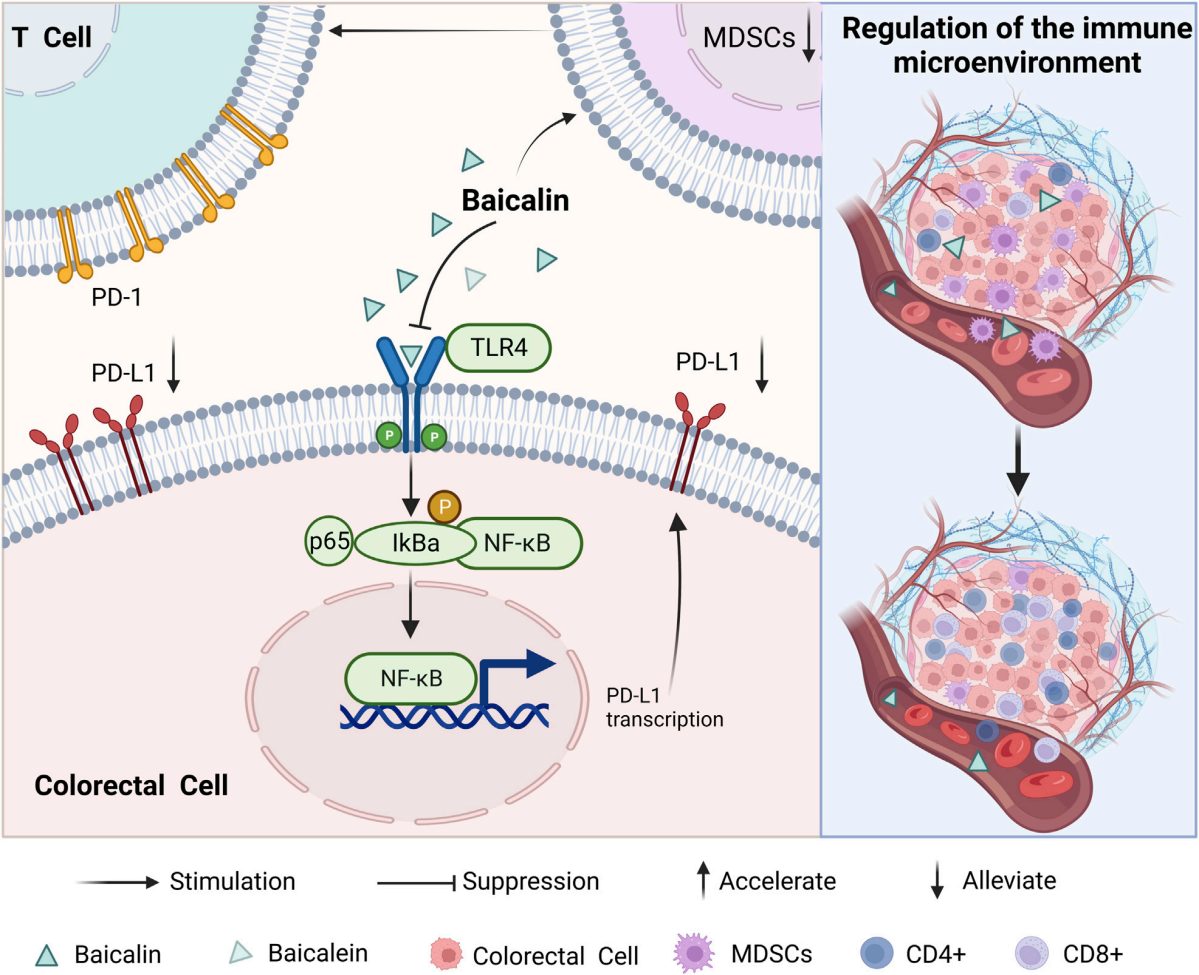
Mechanisms of baicalin and baicalein in regulating the tumour microenvironment.

In individuals with tumors, the immune system experiences suppression, resulting in its inability to effectively recognize and eliminate aberrant cells in a timely manner. It was found that the expression of PD-L1 and the proportion of myeloid-derived suppressor cells (MDSCs) in CRC cells were downregulated after baicalin action. This led to T cell activation and upregulation in the proportion of CD4^+^ and CD8^+^ T cells in the body. Enhancing the body’s immune recognition ability to play a role similar to that of western medicine immunotherapy. The expression levels of upstream regulators TLR4, NF-κB, p65, and p-IκBα were also suppressed. This suggests that baicalin may inhibit the TLR4/NF-κB signaling pathway, thereby reshape immunity in the TME (Song et al., 2022), which has the potential to enhance the performance of anti-tumour immunological drugs. (As shown in Figure 5).

Several previous studies have confirmed that long-term inflammatory stimulation of the body can induce damage to the colorectal mucosa, resulting in abnormal cell proliferation with excessive tissue repair, leading to the development of CRC (Shah and Itzkowitz, 2022). Both baicalin and baicalein have been found to have potential in adjuvant chemotherapy for CRC. Additionally, they have demonstrated the ability to decrease the infiltration of inflammatory factors, thereby inhibiting the progression of colorectal inflammation to CRC. Baicalin reduced the expression of inflammatory factors, including IL-1β, IL-6, and NF-α in tissues, improving local inflammatory infiltration. Conversely, baicalin increased the expression of PPARγ in CRC cells, thereby inhibiting NF-kB activation and reducing the expression of inflammatory factors such as iNOS, COX-2, and TNF-α, ultimately inhibiting the development of inflammatory-cancerous transformation (Kim et al., 2013; Lin H. D. et al., 2023). In another study, baicalein was found to decrease Keapl expression and increase the expression of Nrf2, increasing the antioxidant capacity of the body by regulating the Nrf2 signaling pathway, thus exerting an anti-colorectal cancer effect (Havermann et al., 2016). The above evidence suggests that baicalin and baicalein have a reversing effect on the transformation of CRC, which is expected to change the intervention window for CRC and block the appearance of colorectal inflammatory cancer transformation.

## 3 The use of baicalin and baicalein in adjuvant chemotherapy for CRC

Furthermore, researchers have conducted studies investigating the potential synergistic effects of combining baicalin and baicalein with chemotherapeutic drugs in the treatment of CRC. These investigations aim to explore the ability of these compounds to attenuate the impact of chemotherapeutic drugs on CRC. Baicalin was found to reduce intracellular ROS and mitochondrial superoxide levels and improve antioxidant proteins such as GSH, Mn-SOD, and HO-1 to enhance the antioxidant defense system of CRC cells. Moreover, it regulates the release of pro-inflammatory cytokines IL-6 and TNF-α by inhibiting the Wnt/β-certain signaling pathway to improve the symptoms of peripheral nerve damage caused by oxaliplatin (Jugait et al., 2022). Another common adverse effect of chemotherapy irinotecan is an acute cholinergic syndrome and delayed diarrhea. As early as 1995, Japanese scholars discovered that baicalin, the main ingredient in laxative soup, could counteract the diarrheal effects of irinotecan (Takasuna et al., 1995). Due to the development of microbiological and other histological techniques, baicalin was found to be reduced to baicalein in the human intestine by GUS under the action of intestinal flora (Akao et al., 2000). Furthermore, recent studies have confirmed that selective inhibition of GUS activity can reduce the incidence of irinotecan-induced diarrhea (Chamseddine et al., 2019). Based on the above evidence, it is assumed that baicalin may have reduced the symptoms of irinotecan-induced diarrhea by competitively inhibiting the activity of GUS.

## 4 Discussion

**FIGURE 6:**
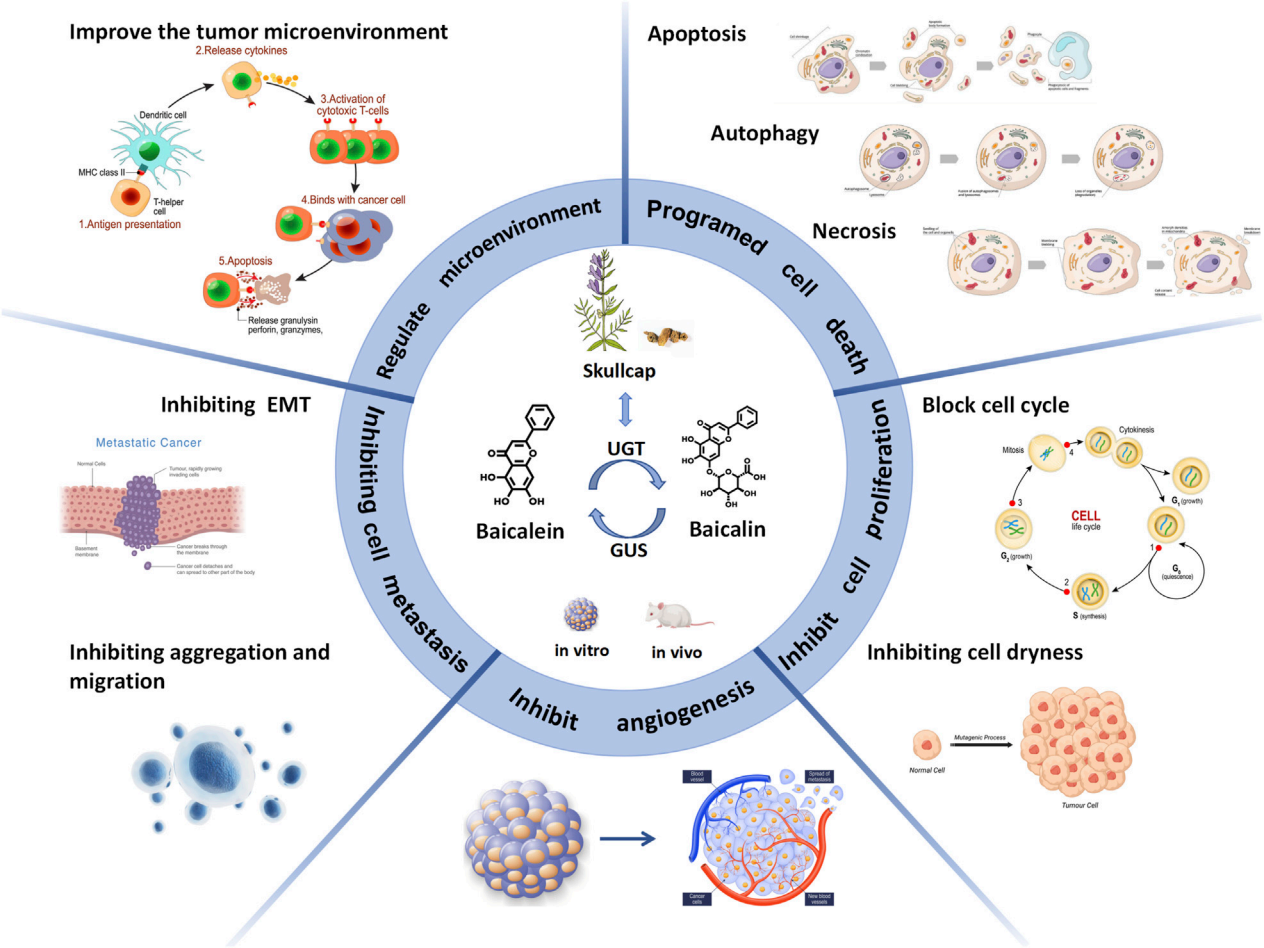
Baicalin and baicalein exert anticolorectal cancer effects by inducing cell death and inhibiting cell proliferation, angiogenesis, cell metastasis and improving the tumour microenvironment.

Baicalin and baicalein have the potential to become new drugs in the clinical fight against CRC. This article comprehensively discusses how baicalin and baicalein exert their anti-colorectal cancer effects through different molecular mechanisms and signaling pathways. Studies have shown that both entities possess anti-colorectal cancer properties through various mechanisms, operating at different stages and multiple levels. These mechanisms encompass the promotion of apoptosis, autophagy, and cell necrosis to induce cellular demise, hindrance of cell cycle progression and stemness to impede cellular proliferation, suppression of tumor angiogenesis, prevention of epithelial-mesenchymal transition and cell aggregation to inhibit invasion and metastasis, and regulation of the tumor microenvironment. Additionally, baicalin and baicalein can be used as adjuvant agents in chemotherapy to reduce the incidence of adverse reactions associated with this treatment modality. Furthermore, their utilization may enhance the suppressive effects on infiltrative, thereby impeding the progression of inflammatory-carcinogenic transformation. (As shown in Figure 6).

In addition to these mechanisms, there is increasing evidence that non-coding RNAs regulate CRC. Circular RNA (circRNA) has emerged as a potential novel therapeutic target for CRC treatment. Some researchers found that circHERC4 can be a novel oncogenic driver to promote CRC tumor metastasis. This effect is achieved through the activation of the miR-556-5p/CTBP2/E-cadherin pathway. Furthermore, the expression of circHERC4 is significantly related to the prognosis of CRC patients (He et al., 2021). A previous investigation demonstrated that the molecular mechanism underlying the impact of Leigongjiang polysaccharide on CRC involves the downregulation of IncRNA PRR34-AS1 expression and modulation of the downstream Foxo3a/Wnt/β-catenin pathway (Mou and Shu, 2023). The tumor microenvironment is the soil for tumor growth and largely influences the evolutionary process of tumors. As messengers between tumor cells and host cells, exosomes are considered key mediators in regulating the tumor microenvironment and are closely related to tumor progression and drug resistance development (Yao et al., 2023). A recent study found that serum exosomes miR-377-3p, miR-381-3p, and miR-6803-5p could be potential biomarkers of CRC (Yan et al., 2018; Wang et al., 2022b). The researchers found that miR-223-3p increased the resistance of CRC cells to 5-fluorouracil by targeting SORBS1, resulting in insensitivity to CRC treatment through transfection (Lin Y. L. et al., 2023). This shows that non-coding RNAs and exosomes impact the development and treatment of CRC. In the future, we will further explore whether baicalin and baicalein can play an anti-colorectal cancer role by interfering with the regulation of non-coding RNAs and influencing exosomes to regulate the tumor microenvironment.

Although there are many studies on baicalin and baicalein against CRC, the key mechanisms remain unclear as they act in a multi-target and multi-pathway rather than a unidirectional manner. The existing studies are mainly *in vitro* cellular and animal studies, which differ from the growth and evolutionary environment of CRC in humans. The constraints that result from the two-dimensional nature of the plane cell assay, which lacks three-dimensional structures and cell-to-cell interactions, as well as the ethical considerations governing animal experimentation, impose limitations on the scope of research. The rise of organoids has compensated for the shortcomings of traditional research models and has recently shown great potential in the study of clinical tumors. In subsequent investigations, organoid utilization modeling may further explore the potential physiological effects of baicalin and baicalein within the human body (Qu et al., 2021).

The bioavailability of a drug is influenced by its absorption and distribution. For example, baicalin and baicalein are flavonoids and exhibit limited bioavailability due to their hydrophobic nature and degradation sensitivity, resulting in unsatisfactory therapeutic effects. Some studies have shown that baicalein is more effective than baicalin against CRC, and the bioavailability of baicalin after oral administration is only 2.2%, which limits its clinical application (Xing et al., 2005). Based on the metabolism of the two compounds *in vivo* and the differences in their molecular structures, it is speculated that the presence of sugar groups on the molecular structure of baicalin may result in lower water solubility *versus* lipid solubility and less access to cell membranes. The intestinal flora has been found to rapidly convert baicalin to baicalein and play an important role in improving blood concentration (Wang et al., 2015). Due to the development of nanotechnology, researchers have found that combining baicalin and baicalein with nano transmitters can significantly increase their bioavailability *in vivo* and enhance their anti-tumor activity (Ibrahim et al., 2022; Zheng F. et al., 2023).Moreover, new formulations such as solid dispersions, liposome formulations, phospholipid complexes, β-cyclodextrin inclusions, and metal complexes provide new technical support to enhance the anti-tumor activity of baicalin and baicalein (Long et al., 2019).

## 5 Conclusion

In this review, we summarize the mechanism of action of baicalin and baicalein against CRC. It was concluded that the two compounds under investigation not only induce cell death and suppress cell proliferation, angiogenesis, cell metastasis, and improve the tumor microenvironment, but they also have the potential to mitigate the adverse effects associated with chemotherapy (Table 1). Furthermore, these compounds can improve the colorectal inflammatory microenvironment, impeding the inflammatory-cancer transformation process. Although the above evidence suggests that baicalin and baicalein have great potential in treating CRC, certain issues persist: For example, the development of CRC involves multiple signaling molecules and is regulated by multiple signaling pathways. However, existing research predominantly focuses on investigating individual molecular mechanisms or specific signaling pathways, lacking a comprehensive understanding of the overall process. Therefore, multiple pathways and levels of association can be undertaken to reduce to some extent the limitations and one-sidedness of understanding CRC in the future. Furthermore, despite the aforementioned evidence substantiating the effectiveness of two drugs in both *in vitro* and *in vivo* animal studies, the extent to which they can effectively combat CRC in humans remains a subject of debate. Additionally, further clinical studies are required to validate the fundamental mechanisms and targets of intervention associated with these two drugs in future research endeavors. However, in general, the therapeutic role of baicalin and baicalein in the field of CRC should not be overlooked and warrants more in-depth studies to promote their clinical translation in the future.

**TABLE 1:** Molecular pathways of baicalin and baicalein against colorectal cancer.

Mechanisms	References	Material	Models	Pathway	Upregulates	Downregulates	Experiment
Induction of CRC cell death							
Apoptosis	Wang et al. (2019)	Baicalein	HT-29 cell	AKT/mTOR	p53	AKT	*In vitro*
					Caspase-3	Bcl-2	
					Bax p53	Wnt	*In vivo*
					PTEN	β-catenin	
	Jia et al. (2019)	Baicalin	DLD-1 cell	Wnt/β-catenin	DKK1	microRNA-217	*In vitro*
			HCT-116 cell			β-catenin c-Myc	
	Li et al. (2021)	Baicalin	SW620 cell	PI3K/AKT/GSK-3β	-	p-PI3K	*In vitro*
						p-AKT	
						GSK-3β	
	Lin et al. (2023a)	Baicalin	SW420 cell	Hedgehog	Caspase-3	SHH	*In vitro*
					Caspase-9	SMO	*In vivo*
					SUFU	Gli1	
	Xu et al. (2022)	Baicalein	SW480 cell	MET/AKT	ROS	p-MET	*In vitro*
					Caspase-3	p-AKT	
					PARP	p- H3	
	Su et al. (2018)	Baicalein	HCT-116 cell	JNK/p38	DEPP	-	*In vitro*
					Gadd45a p-MAPK		
	Tan et al. (2019)	Baicalein	SW1417 cell	PI3K/AKT	Caspase-3	Bcl-2	*In vitro*
			SW480 cell	STAT3	Caspase-8		
			DLD-1 cell		Caspase-9		
			HCT-15 cell		Bax		
			LS-180 cell				
			CCD-18Co cell				
	Dou et al. (2018)	Baicalein	HCT-116 cell	MAPK/p38 ERK1/2	MAPK p38	-	*In vitro*
			HT-29 cell		ERK1/2		*In vivo*
			SW480 cell				
Autophagy	Tan et al. (2019)	Baicalein	SW1417 cell	PI3K/AKT	-	LC3II	*In vitro*
			SW48 cell			Beclin 1	
			DLD-1 cell				
			HCT-15 cell	STAT3			
			LS-180 cell				
			CCD-18Co cell				
Necrosis	Yang et al. (2019)	Baicalin	CT26.WT cell	-	RIP3	-	*In vitro*
Inhibition of CRC cell proliferation							
Blocking the cell cycle	Dou et al. (2018)	Baicalein	HCT-116 cell	MAPK/p38	MAPK p38	-	*In vitro*
			HT-29 cell	ERK1/2	ERK1/2		*In vivo*
			SW480 cell				
	Wang et al. (2019)	Baicalein	HT-29 cell	AKT/mTOR	p53	AKT	*In vitro*
					PTEN		*In vivo*
	Palko-Labuz et al. (2017)	Baicalin	HCT-116 cell	mTOR	4E-BP1	mTOR	*In vitro*
						p70S6K	
						S6 eIF4E	
Inhibition of tumour stemness	Lin et al. (2023a)	Baicalin	SW420 cell	Hedgehog	SUFU	SHH	*In vitro*
						SMO	*In vivo*
						Gli1	
Inhibition of angiogenesis							
	Wang et al. (2019)	Baicalein	HT-29 cell	AKT/mTOR	p53	AKT mTOR	*In vitro*
					PTEN	VEGF	*In vivo*
	Li et al. (2021)	Baicalin	SW620 cell	PI3K/AKT/GSK-3β	-	p-PI3K	*In vitro*
						p-AKT	
						GSK-3β	
	Chen et al. (2021)	Baicalein	HCT-116 cell DLD-1 cell	TLR4/HIF-1α/VEGF	-	TLR4	*In vitro*
						HIF-1a	*In vivo*
						VEGF	
Inhibition of CRC cell metastasis							
	Chen et al. (2012)	Baicalein	HCT-116 cell	MAPK/p38 ERK1/2	MAPK p38	-	*In vitro*
			HT-29 cell		ERK1/2		*In vivo*
			SW480 cell				
	Kim et al. (2013)	Baicalein	HCT-116 cell	NF-κB	-	MMP-2	*In vitro*
						MMP-9	*In vivo*
	Chai et al. (2017)	Baicalein	HT-29 cell	ERK	-	MMP-2	*In vitro*
			DLD-1 cell			MMP-9 p-ERK	
	Rui et al. (2016)	Baicalein	DLD-1 cell	AKT	-	p-AKT	*In vitro*
						MMP-2	
						MMP-9	
Regulation the tumour microenvironment							
	Song et al. (2022)	Baicalin	HCT-116 cell	TLR4/NF-κB	CD4+T Cell	PD-L1	*In vitro*
			CT-26 cell		CD8+T Cell	MDSCs	*In vivo*
						TLR4	
						NF-κB p65	
						p-IκBα	
	Lin et al. (2023a)	Baicalin	SW420 cell	Hedgehog	-	IL-1β	*In vitro*
						IL-6	*In vivo*
						NF-α	
	Kim et al. (2013)	Baicalein	HCT-116 cell	NF-κB	PPARγ	NF-κB iNOS	*In vitro*
						COX-2	*In vivo*
						TNF-α	
						MDA	
	Havermann et al. (2016)	Baicalein	HCT-116 cell	Nrf2	Nrf2	Keapl	*In vitro*

## Abbreviations

CRC, colorectal cancer; HPLC, high-performance liquid chromatography; UGT, glucuronosyltransferase; GUS, glucuronosidase; PCD, programmed cell death; MOMP, mitochondrial outer membrane permeabilization; Cyt C, cytochrome C; TNF, tumor necrosis factor; TEM, transmission electron microscopy; VEGF, vascular endothelial growth factor; EMT, epithelial-mesenchymal transition; TME, tumor microenvironment; MDSCs, myeloid-derived suppressor cells; circRNA, Circular RNA.
